# Application and Empirical Verification of the Archard Model in the Deburring Process

**DOI:** 10.3390/ma18102387

**Published:** 2025-05-20

**Authors:** Karol Falandys, Krzysztof Kurc, Jacek S. Tutak

**Affiliations:** Faculty of Mechanical Engineering and Aeronautics, Rzeszow University of Technology, al. Powstancow Warszawy 8, 35-959 Rzeszow, Poland; k.falandys@prz.edu.pl (K.F.); tutak_j@prz.edu.pl (J.S.T.)

**Keywords:** wear, optimization, Archard model, deburring

## Abstract

This paper presents a method of numerical simulation, using the finite element method for the brush wear process during the deburring of the edge of the workpiece. The work was carried out in the Ansys Workbench environment in the Ansys Mechanical module. This study reviews the effect of selected parameters of the technological process (rotational speed and depth of tool penetration into the workpiece) on the abrasive wear of the tool. The discussion examines the subject of the 3D or 2D approach in terms of results, quality, and time of computation. A series of numerical analyses (2D) were carried out to investigate the effect of process parameters on the wear rate and, consequently, on the tool life. Obtained results on the quantity of worn material were critically assessed in relation to real-world industrial conditions. The difference between the numerical model and the test performed in the industry environment varied from 3 to 46% and was discussed in this paper. Additionally, to improve the quality of the results in Ansys, an APDL script with adaptative mesh was prepared. The article contains a discussion on the possibility of numerical model development.

## 1. Introduction

Manufacturing companies strive to minimize the costs associated with the production of products, for example, by focusing on the automation of production operations or by optimization of tool usage. In addition, especially those operating in the aviation or space industry strive to maintain the appropriate level of quality of manufactured parts while reducing costs. These tasks are assigned to multidisciplinary teams consisting of automation engineers, technologists, designers, and FEM analysts. Meeting all of the above goals at the same time is a very demanding task, and the process of implementing solutions is time-consuming. One of the methods that allow accelerating research and development work without losing the quality of the results is the use of numerical simulations based on the finite element method (FEM). Numerical analyses have found wide-scope application in industry due to their numerous advantages. For example, the use of numerical models leads to cost reduction by shortening the prototyping phase. Also, the ease of introducing design changes, e.g., modification of friction coefficients resulting from different methods of preparing cooperating surfaces, has a positive impact on the project budget and timeframe. Comparing simple aspects, such as the amount of space needed for analysis versus the amount of space needed for testing, numerical analysis is definitely more economically advantageous. Thanks to this, FEM analysis has gained wide application in almost all sectors of industry. Nowadays, it is almost unimaginable to build a commercial aircraft, a car for a wide range of customers, or a windmill that generates electricity on windy days without using numerical simulations. Due to the numerous and indisputable advantages resulting from the implementation of FEM simulations, it was decided to use this method to investigate the effect of selected parameters of the brushing process on the service life of the tools used in that process.

Due to the market situation (price competition) and economic conditions (European Union “Fit for 55” policy), the vast majority of production plants aim to rationally use available resources. Those resources take the form of electricity, the number of working hours necessary to produce a given part, or the number of tools used in the production process. In recent years, as electricity and tool prices have increased, aviation companies have intensified their efforts to reduce energy and consumables’ consumption. These efforts have a positive impact on companies’ financial results (reduction in the cost of producing parts) and lead to a very significant reduction in the carbon footprint (reduction in CO_2_ emissions).

There are a number of possibilities to achieve the above-mentioned goals related to both financial savings and environmental protection. In order to reduce the number of working hours, solutions using automation and robotization of individual stages of the production process can be implemented [1,2,3,4,5,6,7,8,9]. By introducing automation and robotics into the production process, the repeatability of the production process is also increased, which in turn allows for optimization related to the use of tools. Robotic welding enhances structure quality and production. Future research aims to overcome challenges by expanding material use and advancing automation [1]. Ornat et al. (2022) presented a study on the development of a robotic geometry inspection station for ADT gearbox housing using laser profilometers, emphasizing the importance of precise quality control in the aviation industry, where optical scanners aid in measurement before, during, and after processing [2]. Kurc et al. (2022) explored the application of a 3D scanner in robotic measurement of aviation components, showcasing advancements in measurement technology for aerospace manufacturing [3]. Glukhov et al. (2019) analyzed the functional capabilities and features of information system software components for collecting, processing, and ensuring the reliability of aircraft technical and resource data [4]. Wu et al. (2014) introduced a novel material removal model, analyzing robot velocity and contact force using a superposition method for faster pressure distribution estimation in robotic grinding processes [5]. Burghardt et al. (2017) presented a robotic test stand for ultrasonic tomography inspection of stator vane thickness, further enhancing the quality control process in aerospace manufacturing [6]. Hetmanczyk (2024) proposed a method to assess robotization maturity in manufacturing amid digital transformation, aligned with Industry 5.0 principles [7]. A survey of 200 Polish SMEs (2022–2024) evaluated automation, digitization, flexibility, and data integration to enhance digitalization and future development [7]. Finally, Sha et al. (2023) introduced an automated system using fine-tuned YOLOv5 models for accurate solder joint inspection on aviation plugs, advancing the application of AI in quality control [8]. Bernabei et al. (2023) presented a framework to assess the smart level of data-driven processes in aerospace manufacturing, guiding companies toward greater digitization [9].

The introduction of automation and robotics also resulted in improved working conditions by limiting the impact of harmful factors, such as noise and dust, on company employees, as well as eliminating the risk of injuries [10,11,12,13,14]. The survey in [10] examined safety in human–robot interactions, focusing on industrial robots, autonomous mobile robots, and assistive robots. It highlighted potential hazards, risk assessment, and the impact of autonomous vehicles on road safety. Industrial robots [11] pose injury risks during maintenance and setup. The paper explored accident causes, risk assessment methods, safety strategies for collaborative robots, and a case study on an industrial robot in injection molding, summarizing risk analysis and mitigation measures. The study in [12] assessed the robot palletizing operation at XYZ Manufacturing using the ANSI/RIA R15.06 risk methodology. The authors of [13] stated that the robot used in developing regions, like Guangdong, China, initially increased occupational injuries, but this declined over time. The article in [14] reviewed literature on robot safety and reliability, categorized into reliability, safety and maintenance, and general topics. However, a common feature of all the above-mentioned activities related to the implementation of new production solutions is the need to test them before the implementation stage.

The testing phase of the project is time-consuming and expensive, but it allows to eliminate the weaknesses of the proposed solutions, which results in significant savings of time and resources in the later phase of the project. One of the methods that allows for reducing the number of tests is computer simulations based on the finite element method (FEM) [15,16,17,18,19,20,21]. The use of a virtual environment allows for significant savings in time and other resources necessary for preparing tests. For example, instead of blocking the machine with the organization of process parameter tests, you can prepare an appropriate simulation and obtain a pre-optimized set of parameters, which should be confirmed in reality.

While FEM-based wear analysis is widely used in industrial applications for simulating machining and finishing processes, the current study presents a distinct contribution. Specifically, it focuses on the abrasive wear modeling of silicon carbide (SiC) bristle brushes during the deburring of nickel-based turbine blades—an application scenario not covered in existing open-access literature. Previous studies have examined SiC in different contexts. Tsai (2024) explored abrasive jet machining of nickel 200 with SiC and Al_2_O_3_ particles using a microcontact wear model, but without FEM analysis or brush-based deburring [22]. Ji et al. (2015) conducted FEM simulations of microgrinding with SiC as the workpiece material, rather than as an abrasive acting on nickel-based alloys [23]. Similarly, Bushlya et al. (2013) studied SiC-whisker-reinforced cutting tools in high-speed machining of Alloy 718, but this involved cutting—not brushing—and did not utilize FEM wear modeling [24]. In contrast, the current work develops and validates a FEM wear model based on the Archard law, correlating simulation outcomes with empirical data obtained from a real industrial deburring process. This integrated approach not only addresses a gap in the literature but also provides a foundation for future optimization and digital twin applications in surface finishing of high-performance aerospace components.

In line with the above trend, work was undertaken to understand the trend of modifying the depth of the tool’s approach to the workpiece on the nature of tool wear. In the work in [25] related to the deburring process, it was observed that modifying selected parameters of the technological process, such as the depth of approach or rotational speed, affects the level of moments and forces interaction between the brush and the workpiece. Taking into account the need to find a local optimum that guarantees the most effective tool wear, it was decided to prepare a numerical model simulating the edge dulling process. The model will consist of a tool (brush) and a part of the workpiece that is supposed to represent the actual geometry of the workpiece. Due to the complexity of the issue, the way tools wear out, and the availability of computing power, it was decided to conduct a two-dimensional (2D) analysis. The aim of preparing the numerical model is to determine the influence of the selected parameter of the brushing process [26] on the way the tool wears (abrasions). In this way, the results obtained from the tests will be verified, and then the validated model can be used for further optimization work related to, for example, changing the tool material. By conducting numerical simulations, the number of tests necessary to be performed in the production environment will be reduced, which will lead to a reduction of time and costs of implementing new solutions aimed at extending the tool’s service life (cost reduction and carbon footprint reduction). In the publicly available literature, one can find information that the topic of abrasive wear simulation using the Archard model is the subject of research by many authors. They have conducted numerical simulations in order to determine and optimize the abrasive wear of tools [27,28,29].

## 2. Materials and Methods

**Figure 1:**
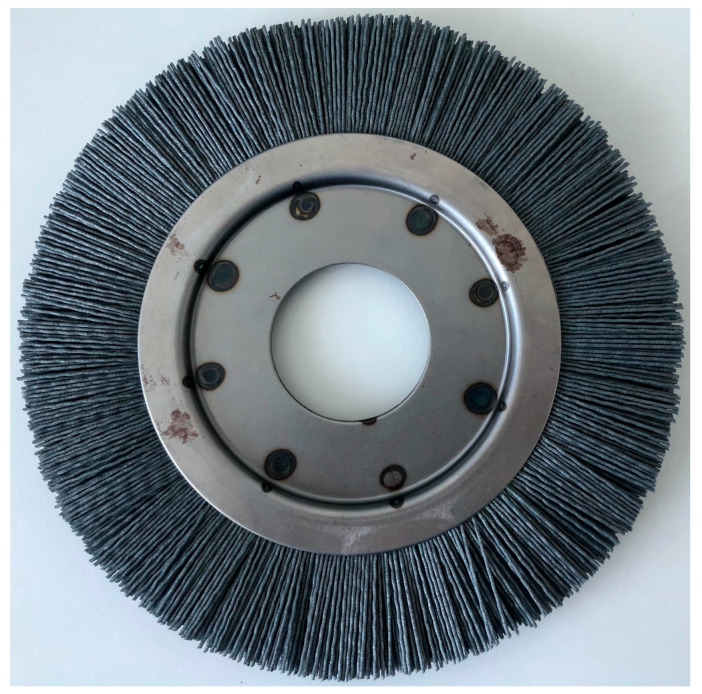
A tool—a brush—used in the deburring process.

One of the key aspects of business activity in the production area is the unit cost of producing a given detail. The vast majority of companies operating in economic competition aim to increase profits by reducing production costs. There are a number of methods that allow to achieve the desired effects in terms of reducing the above-mentioned costs, such as searching for markets with low labor costs, improving the quality of the products offered, or optimizing production processes. In the case of striving to improve the efficiency of the production process, the above-mentioned cost is influenced by many factors. Possibilities for improvement can be found in the reduction of production operations, the level of process robotization, energy consumption, or the quantity and parameters with which individual tools are used. Due to the limited repeatability of production operations performed by operators, only those operations that have previously been automated and robotized can be subject to advanced optimization. Then, projects related to ensuring the optimal use of tools and production resources in terms of finance and quality can start. The article in [26] presents a selected methodology aimed at extending the tool life (in the form of a rotating brush) during the edge deburring and burr removal process. The work carried out led to a significant reduction in tool wear, which had a positive impact on the financial result (reduction of unit costs) and led to an improvement in the rational use of resources (reduced tool consumption reduced the carbon footprint). Due to limited resources in the form of machine usage time and the number of available tools and parts, the tests conducted so far have covered only the most promising range of technological parameters. The aforementioned range was developed based on many years of experience of the production and engineering team. Taking into account the costs necessary to carry out the tests (machine time, tool costs, parts costs, and personnel man-hour costs) and striving to further reduce production costs, it is considered important to carry out further research work aimed at verifying the correctness of the tests carried out in a virtual environment. It is also important to verify the obtained parameter relations in a much wider range of input parameters (depth of the workpiece’s approach to the tool). The authors of this study aim to verify the relations that link the input parameters in the form of the depth of the workpiece’s engagement to the tool to the degree of bristle wear. This relationship will be examined in two parameter ranges. The first will be the range used for the work carried out so far, and then verification of whether the obtained relationships are true for a wider range of parameters. In this way, confirmation will be obtained that the proposed technological parameters are optimal in terms of tool wear. The construction of a numerical model aimed at stimulating abrasive wear will have a positive impact on the costs. The expenses of developing technological parameters by limiting the use of the workstation, the number of used tools, or by limiting the staff resources necessary to perform the verification are reduced. In order to verify the influence of technological parameters on the tool service life (Figure 1), it was decided to prepare a numerical model based on the finite element method in the Ansys Workbench Mechanical environment.

**Figure 2:**
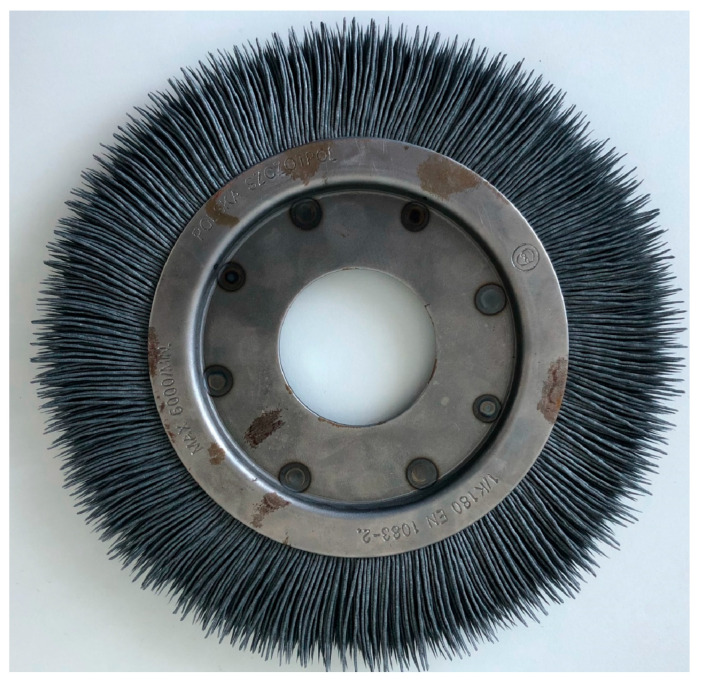
Brush in worn condition.

One of the most important steps before starting to develop a numerical model of the analyzed phenomenon is to decide whether to implement the model in a two-dimensional or three-dimensional environment. Based on engineering practice and experience in the implementation of the edge dulling process in industrial large-scale production, it was decided to observe the way the bristles wear out. Due to the performed observation of the way tools wear out, it was decided to prepare the model in a two-dimensional environment. Observations clearly showed that the bristles wore exclusively in the direction of sliding (tangential and circumferential direction of the tool) on the machined surface. Figure 2 shows a typical condition of the bristles for a worn brush (used in the process).

Based on the measurements taken and their comparison with the initial state, there was no abrasion of the bristles in the axial direction of the brush. Contact between bristles ceased to be important. In connection with the above, the issue of tool–part contact can be simplified to a two-dimensional issue.

Another simplification used in the construction of the numerical model is the representation of the processed detail by a simple rectangle. This shape reflects the cross-section of the workpiece processed in the actual deburring process. The simplification did not negatively affect the quality of the obtained results, because the prepared model was not used to verify the state of the deburred edges (their dimensions). The main goal of building this model was to understand the wear of the abrasive tool during the process of finishing the outer edges (abrasion of the bristles). The subsequent part of the article presents the individual steps of building the numerical model.

**Figure 3:**
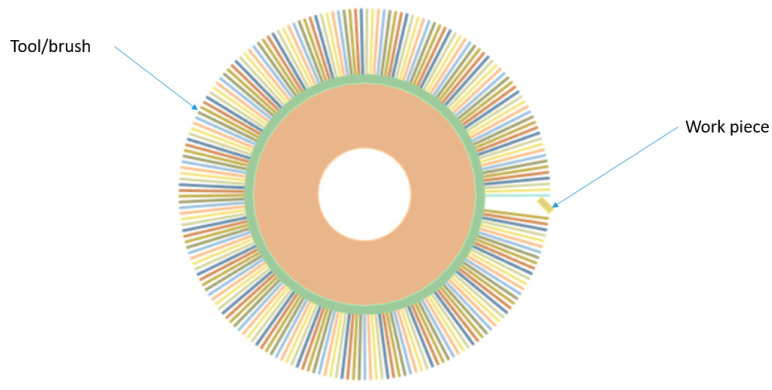
Prepared geometry.

The numerical model consists of the following elements: a disc with a bristle and a rectangle (a cross-section through the workpiece) simulating the workpiece being machined. All the geometric elements included in the numerical model are shown in Figure 3.

Based on the technical documentation included with the Ansys Mechanical software (V22R02-05), the simulation of abrasion occurring in the process where at least one of the bodies had a rotational speed could be reduced to a quasi-static analysis. Material data in the form of Young’s modulus and Poisson’s ratio are presented in Table 1.

**Figure 4:**
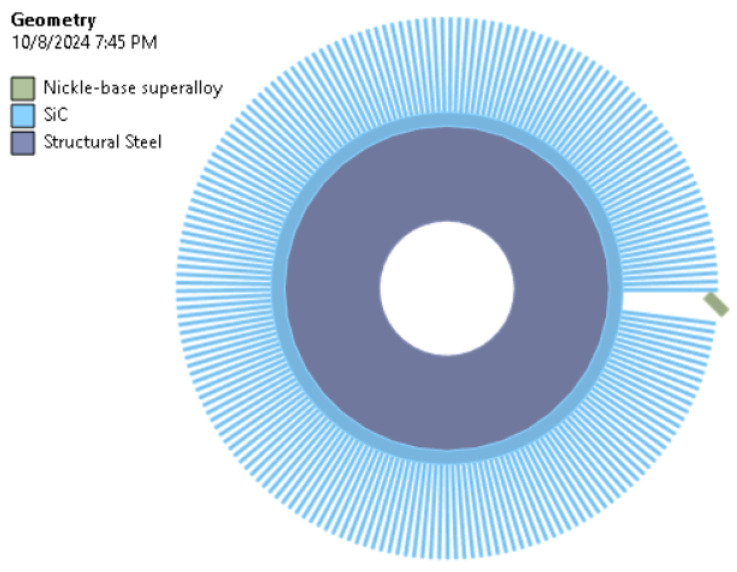
Assignment of material properties.

The assignment of individual materials to the appropriate bodies is presented in Figure 4. The brush was divided into two bodies, where the inner ring was made out of steel, while the bristles were made of silicon carbide.

**Figure 5:**
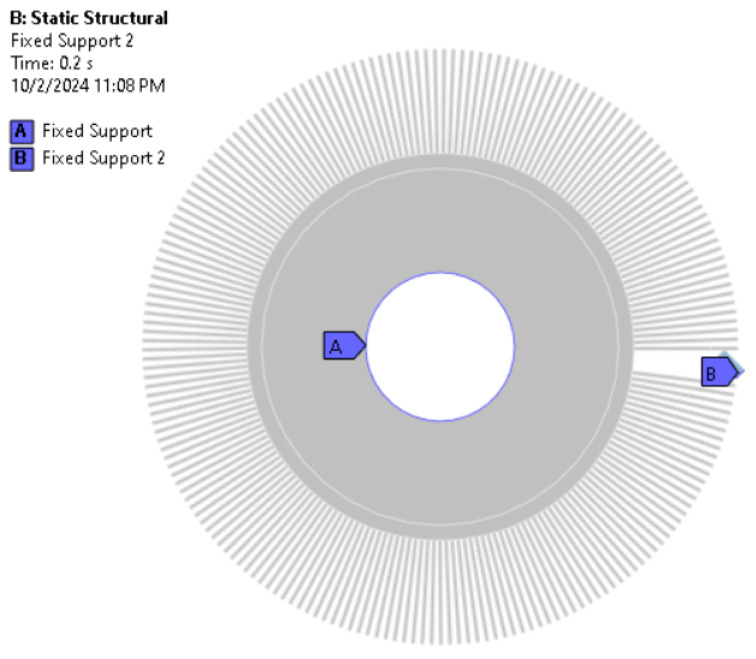
Boundary conditions.

Taking advantage of the possibility of using quasi-static simulation, the boundary conditions in Figure 5 were introduced in the form of fixed supports.

**Figure 6:**
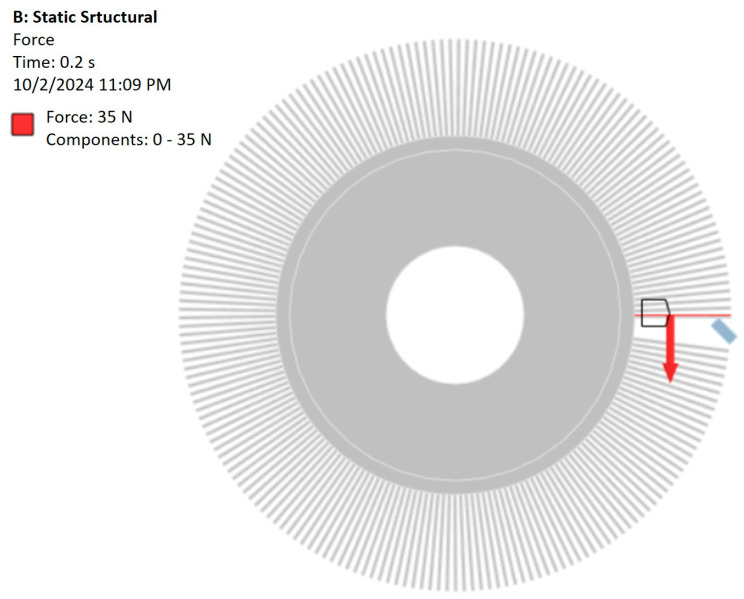
Force applied to the model.

In order to take into account the mutual influence of the tool and the workpiece, it was decided to apply the force that was measured during the tests. Due to the small variation of the force, it was decided to simplify the modeling by adopting the average value determined for the extreme values of the parameters related to the rotational speed and the depth of the workpiece engagement to the tool. The force value was 35 N [26]. The force was applied to the surface indicated in Figure 6, in accordance with the presented reference system.

**Figure 7:**
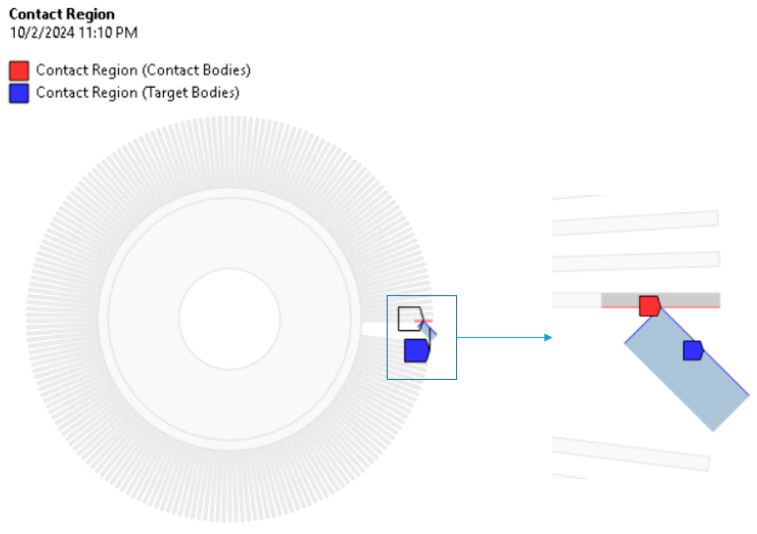
Contact pair.

In order to correctly model the mutual interaction of bodies presented in the model, a contact pair was defined according to the details in Figure 7.

The necessity to take into account abrasion simulation by the Archard model required, in accordance with the requirements presented by the Ansys software manufacturer, the introduction of a number of specific contact settings [33], such as:

- Augmented Lagrangian formulation,
- Contact stiffness is updated at each iteration,
- Location of the contact detection point: nodal point, normal to target surface.

The issue of abrasive wear was decided to be simulated using the Archard equation. The Archard equation is a model used to describe sliding wear of interacting elements and is based on the roughness theory. The cited abrasive wear model is based on the assumption that the amount of material removed as a result of abrasive wear is proportional to the work done by friction forces. There are a number of scientific studies confirming the validity of the above equation [34,35,36,37,38]. In some sources [39], one can encounter the term Reye–Archard–Khrushchev wear law. The Ansys Workbench environment with the Mechanical module enables the simulation of abrasive wear using two approaches. The first one is the Archard abrasion model, and the second one can be freely defined by the software user. In this work, it was decided to choose the first of the possible models. The Archard model is defined by Equation (1):

(1) $$Q=\frac{K\times W\times L}{H},$$

Q—total volume of worn material,
K—dimensional constant,
W—normal force,
L—slip distance,
H—material hardness (the smallest value of a pair of contacting materials).

In Ansys software, the constants in Table 2 must be defined in order to calculate the abrasive wear.

The wear factor K can be scaled to simplify modeling. As an example, consider the contact of a rotating tool with a stationary workpiece, where the tool rotates at a constant speed. The only effect of this rotation/sliding on the contact surface is to produce wear. The wear factor K can be scaled so that the rotation is not explicitly modeled, but its effect is included in the wear calculations. This significantly reduces the simulation time and effort. More precisely, the wear factor K can be scaled by the rotation speed. In this model, the sliding speed is 2πN*R, where N = 1400 rpm and R is the distance from the axis of rotation. Scaling K by 2πN*R makes the wear rate linearly dependent on the sliding speed without explicitly modeling the sliding [33]. The material constants in Table 3 were defined in the prepared numerical model.

**Figure 8:**
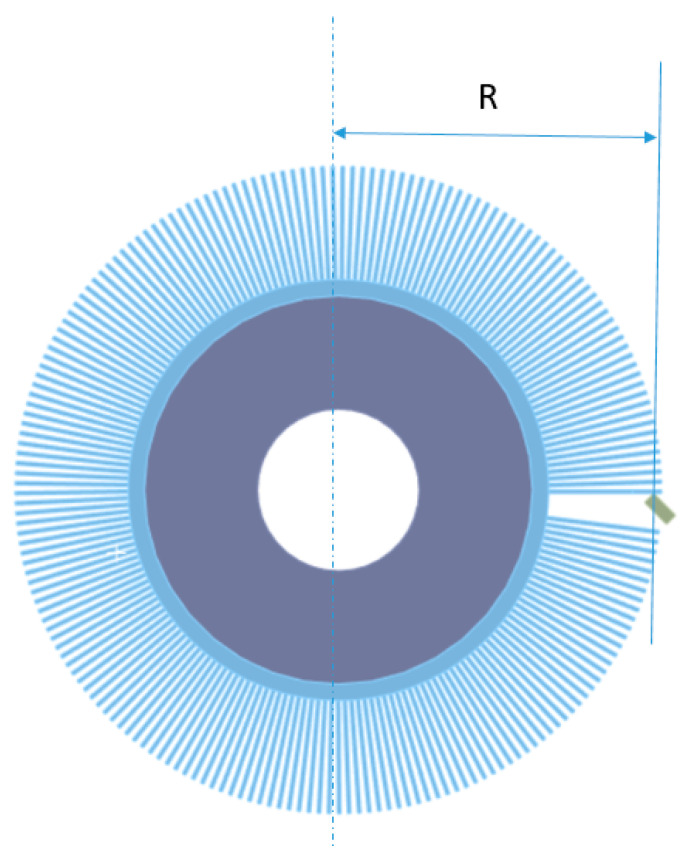
Scaling factor R—the distance from the center of the tool to the point of contact between the bristles and the workpiece.

The values of the scaling factor R (Figure 8) corresponding to the model contact point between the bristles and the workpiece are presented in Table 4.

**Figure 9:**
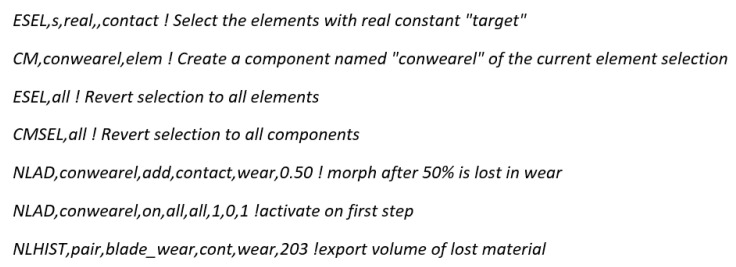
Script.

In order to improve the quality of the obtained results, it was decided to implement a script prepared in APDL (Ansys Parametric Design Language). The script aims to activate adaptive mesh regeneration in the model depending on a specific condition. Mesh regeneration results in improving the quality of the obtained results and has a positive effect on the convergence of the solution. At the end of the script, there is a command, NLHIST, to track the amount of material removed in each calculation iteration. The script is presented in Figure 9 and described in detail.

In the model prepared in this way, it was decided to modify the depth of insertion of the workpiece into the brush in order to verify the influence of the mentioned parameter of the technological process on the modification of the amount of abraded material.

## 3. Results

**Figure 10:**
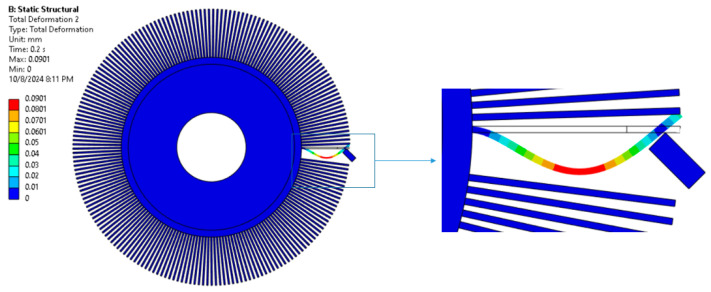
Total displacement, rotational speed 1400 rpm, and insertion depth 4.5 mm.

An example solution for a selected pair of parameters is shown in Figure 10.

**Figure 11:**
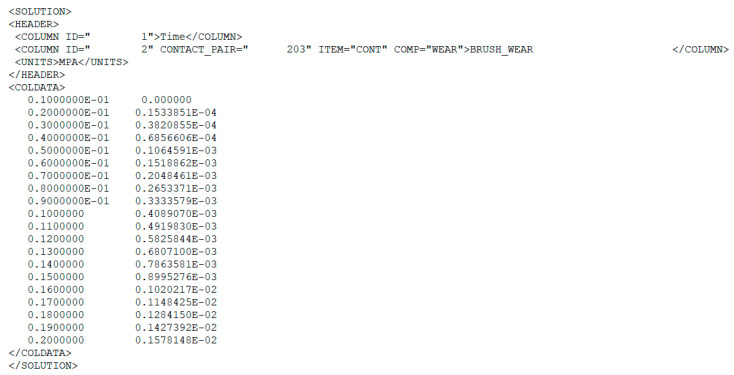
Amount of material abraded, rotational speed 1400 rpm, and depth of penetration 4.5 mm.

As a result of the application of the NLHIST command in Figure 9, the software provided the amount of material worn off in each iteration of calculation. An example of such results is shown in Figure 11.

The results in the form of the amount of material abraded (from the brush bristles) for individual penetration depths ranging from 2 to 8 mm and a rotational speed of 1400 rpm are presented in Table 5. The duration of the analysis was 50 s, which corresponds to the approximate average value for the parts on which the tests were carried out.

**Figure 12:**
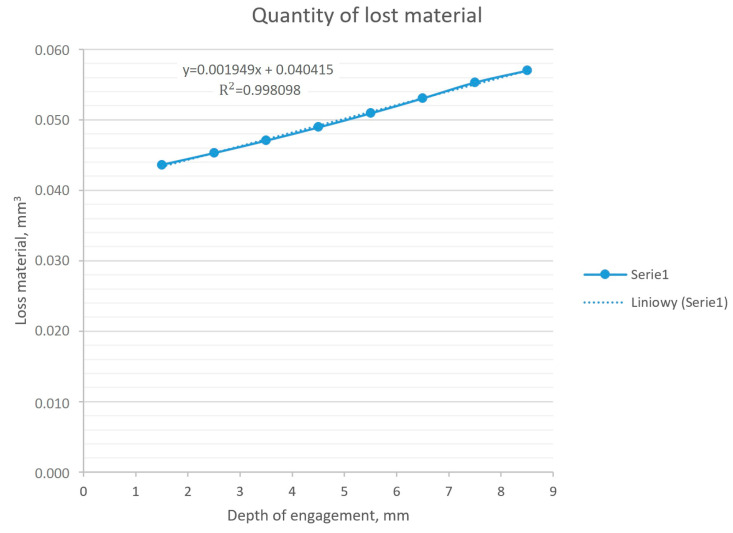
The amount of material removed depends on the depth of engagement, constant rotational speed, and results from the numerical model.

The results presented in Table 5 and their graphical presentation in Figure 12 indicate a linear relationship between the depth of insertion of the workpiece into the tool and the volume of the abraded material.

**Figure 13:**
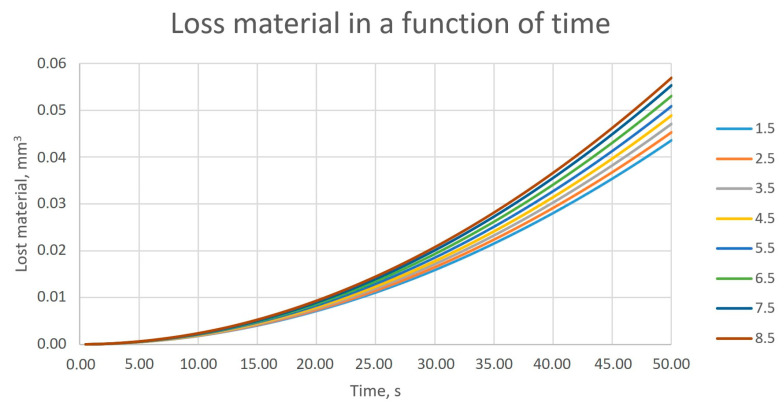
Amount of material removed from 0 to 50 s for individual depths of engagement of the workpiece into the tool, at a constant tool rotational speed.

When analyzing the amount of material worn off depending on the duration of the process, one can notice the nonlinear nature of this relationship (Figure 13). The observed nonlinear behavior of material lost in the time domain can be explained by a change in the contact area. At the beginning, the contact is represented as contact between points and lines, while with the progressive removal of the material of the bristle, it is changed to line to line contact.

## 4. Discussion

In order to determine the influence of the relationship between the depth of insertion of the workpiece into the tool, a series of numerical analyses were carried out, where the above-mentioned parameter was modified. The result of the series of analyses was the amount of material that was worn away in 50 s, which corresponded to the approximate length of the operation in production conditions. Based on the obtained results, it can be stated that this relationship was linear. The calculated coefficient of determination (R^2^) for the obtained dataset was 0.998, which means a linear nature of the relationship. The resulting deviation may result from errors in numerical calculations.

The relationship between the amount of material removed and time was nonlinear, and this was observed for all values of the workpiece insertion depth considered in the study.

### Comparison with Experimental Results

**Figure 14:**
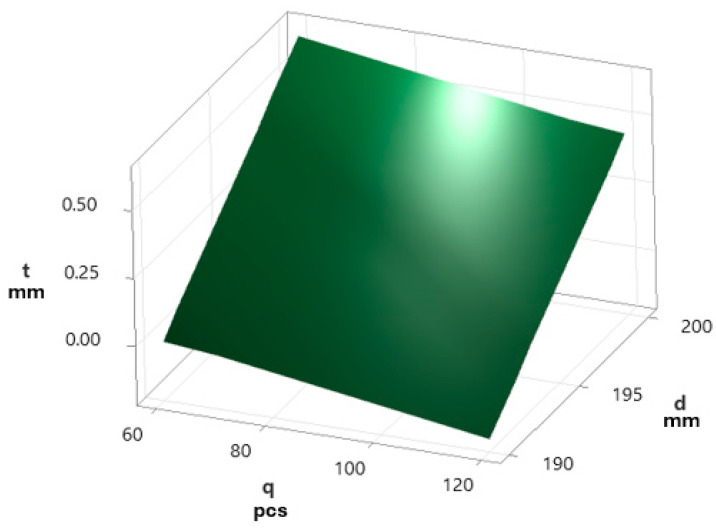
Graphical presentation of Equation (2) (t—bristle thickness in mm, d—brush diameter in mm, and q—number of processed pieces) [26].

As part of the work on selecting the optimal parameters of the technological brushing process (i.e., such parameters that, while maintaining the desired quality of the produced parts, translated into the longest possible brush operation time), a linear relationship was observed between the depth of the part’s insertion into the tool and the amount of abrasive material worn (reduction of bristle thickness). During the test phase of selecting technological parameters, all technological conditions, like used workstation, used tools, workpieces, and environmental conditions, were exactly the same as during standard production process. With respect to this fact, all test results in this paper represent industrial results of deburring technological operation, which is one of the stages of the real production process. The results published in [26] are presented in Figure 14:

(2) t=0.061458×d−0.00288×q−11.5.

An analogous effect of the depth of the workpiece insertion into the tool was observed in the results obtained based on the prepared numerical model. In the case of tests carried out on a robotic station, the brush diameter was controlled as a parameter describing the workpiece insertion into the tool. Both the tests and the numerical calculations confirmed the linear translation of the insertion value on the abrasion of the bristles.

**Figure 15:**
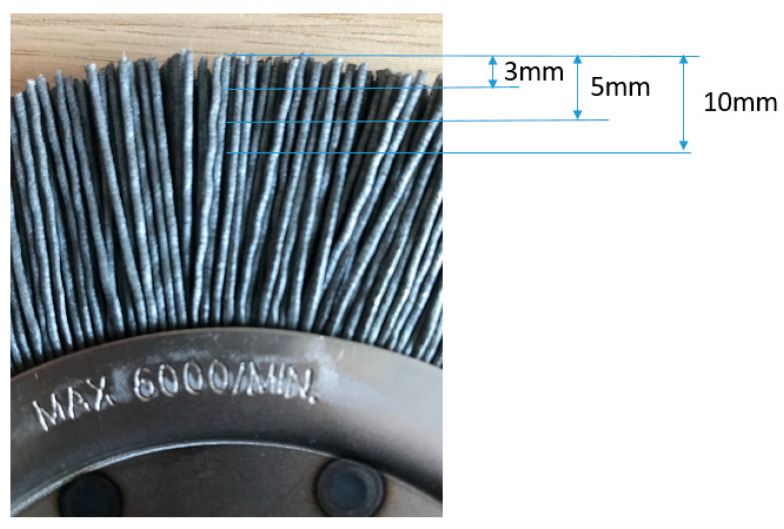
Schema of thickness measurements.

Conducted tests allowed to collect information on the bristle thickness during operation. Measurements were taken in three bristle sections (Figure 15).

**Figure 16:**
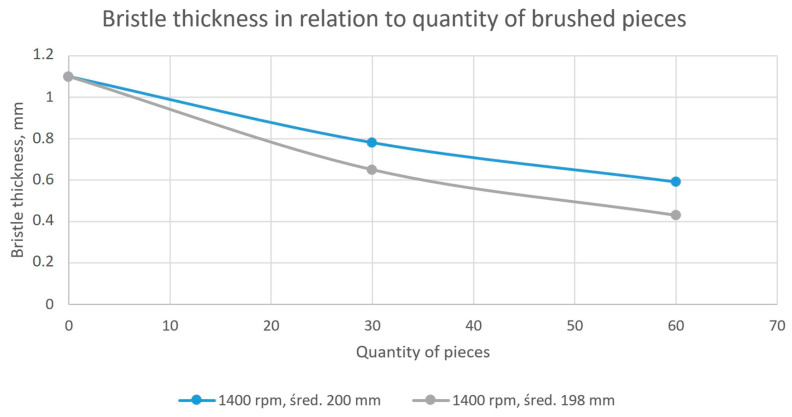
The thickness of the bristles as a function of the selected parameters after working 30 and 60 pieces.

The values for the middle measurement (distance 5 mm from the outer diameter of the brush) after working 30 and 60 blades are presented in Figure 16.

The results of the brush bristle thickness measurements are presented in Table 6.

**Figure 17:**
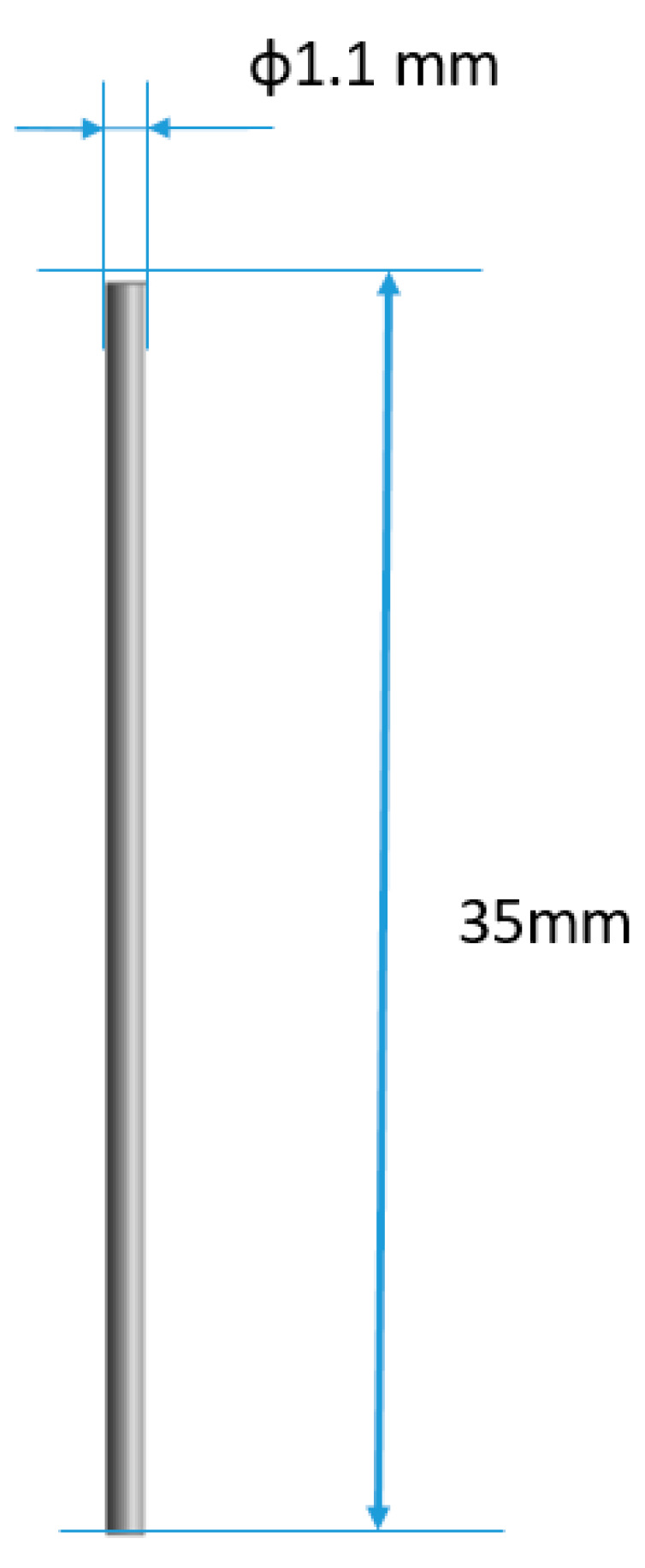
Nominal dimensions of a bristle.

In order to correlate the test results and the numerical model, it was decided to calculate the volume of a single bristle and relate this value to the amount of material removed during the numerical analysis. The nominal dimensions of the bristle are shown in Figure 17.

The nominal volume of a single bristle was 33.26 mm^3^.

**Figure 18:**
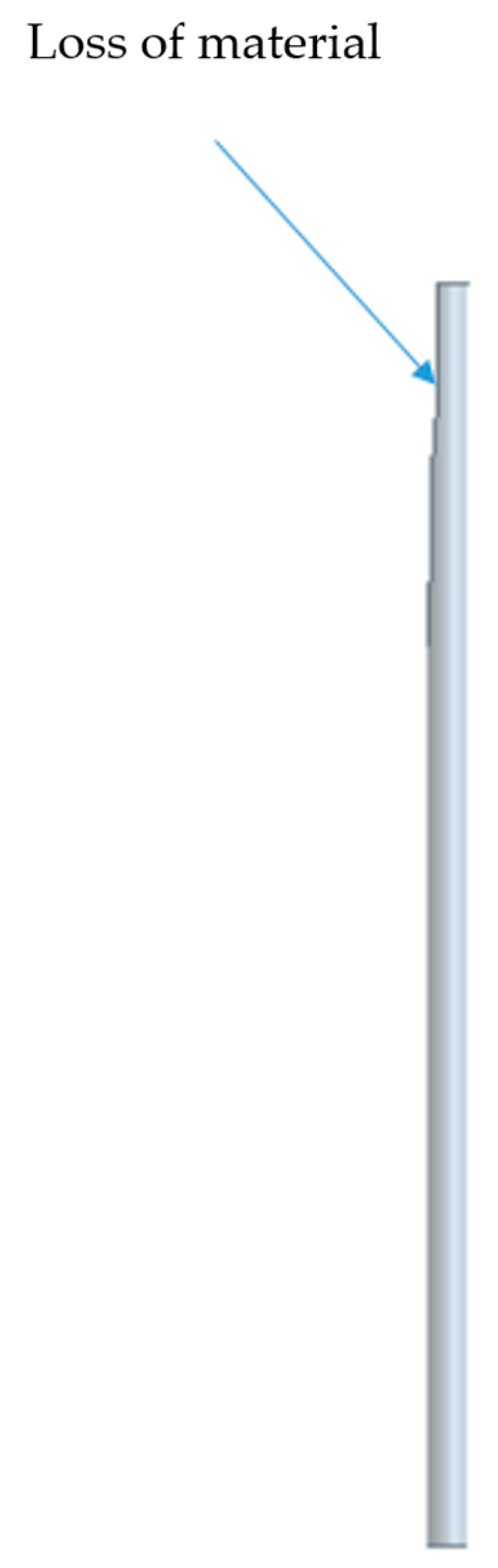
Worn bristle—example.

Four CAD models were prepared, taking into account the data from Table 6 in order to determine the amount of material removed. Figure 18 presents a selected example with the marked zone of material loss.

Table 7 shows the volumes of bristles worn for each test and calculates the average amount of material used. Table 7 also contains a reference to the results obtained using the FEM model.

**Figure 19:**
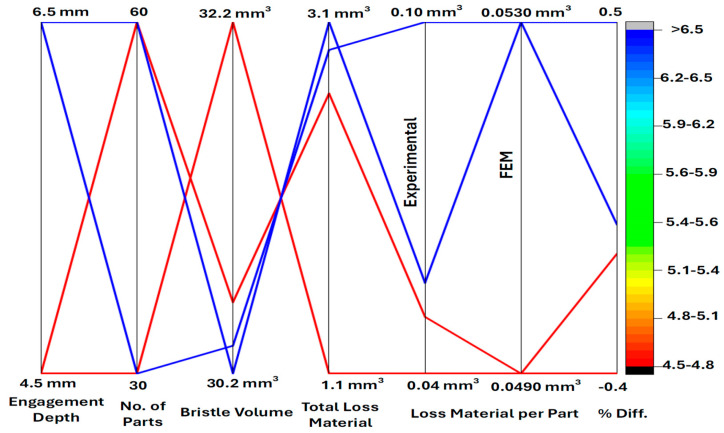
Comparison of single-bristle volume loss between experimental measurements and FEM simulations.

Figure 19 provides a graphical illustration of the data presented in Table 7, further aiding in the comprehension of the differences and correlations between the experimental and simulation results.

The observed differences for measurements concerning brushes that have worked for 30 pieces ranged from 39 to 46%. The observed differences for measurements concerning brushes that have worked for 60 pieces ranged from 3 to 10%. Considering also the graphical representation of the results (Figure 12 and Figure 15), it can be concluded that the material abrasion process itself was nonlinear in nature as a function of time. However, these differences decreased with the extension of the analyzed period (the number of processed pieces). Differences ranging from 3 to 10% of the discrepancy between the test and the numerical model can be considered as correct. From an industrial application point of view, there were no negative consequences due to poor correlation between the FEM model and production process for a group of 30 pieces. The aim of this model was to obtain confirmation about the impact of selected process parameters on the nature of wear. For both, it was confirmed that the depth of engagement had a linear impact on the tool wear ratio. This conclusion allowed us to make a statement that the research described in [26] does not require additional test points.

## 5. Conclusions

Thanks to the observations and conclusions made, the modeling method and the simulation length were significantly shortened, without the need to reduce the quality of the obtained results. The calculations carried out in the virtual environment confirmed the relationships observed during physical tests:

- A linear effect of the depth of insertion of the workpiece into the tool on the amount of abraded material was observed both in the case of the tests performed and the results obtained on the basis of the numerical model.
- A nonlinear relationship between the duration of the process and the amount of removed (worn) bristle material (reduction of bristle thickness) was observed for both the tests and numerical simulations.
- It was observed that the correlation between tests performed in the industrial environment and simulations for the amount of material removed for the 30-piece sample was low (39–46%), while the correlation was high for the 60-piece sample (3–10%).

Based on the above comparison, it can be stated that the numerical model simulating the phenomenon of abrasive wear of the bristles in the brushing process was prepared correctly. The numerical simulation of the abrasion process using the Archard model was relatively easy to implement. This model presented an important feature, namely, the ability to ignore the tool rotation speed and perform quasi-static analysis without explicit modeling of the rotational speed. Scaling the abrasive wear coefficient by multiplying it by the rotational speed significantly simplified the model and, consequently, shortened the calculations.

Thanks to the numerical model, which has been verified by a series of tests, it is possible to continue work on the optimization of the brushing process in the Ansys Workbench environment. By performing numerical simulations based on the finite element method, the execution time and costs of tests will be significantly reduced. As further areas in which solutions should be sought that have a positive impact on the extension of the brush life, it is necessary to indicate work aimed at investigating the increase in bristle thickness for the length of the tool life, or modification of the material from which the bristles are made and verification of the tool life. While a satisfactory correlation has been achieved between the industrial and numerical simulation results, there is still room for further refinement. The authors are planning to enhance the accuracy of the numerical model by developing a digital twin of the deburring process. This model will incorporate the evaluation of temperature and other environmental factors to improve the correlation between the results. The authors place the greatest hope for improving the accuracy of the numerical model and correlation with industrial tests in examining the process temperature. The temperature to which the tool is heated is estimated to be about several dozen degrees Celsius, whereas the temperature of the workpiece is much lower. The temperature of the workpiece during the deburring process is assumed as so low that there is no impact on the microstructure of the workpiece, hence there is no impact on material properties, and thus there is no need to be controlled. However, the temperature of the working tool may lead to changing the stiffness of the bristles and thus have an impact on the numerical model accuracy. In further work, it also seems interesting to examine the bristles from the point of view of breakage and fatigue [42,43].

## Figures and Tables

**Table 1:** Material properties with assignment [30,31,32].

Material	Young Modulus, MPa	Poisson Coefficient	Part
Structural steel	200,000	0.30	Brush inner ring
Silicon carbide (SiC)	410,000	0.15	Brush bristles
Nickle-based superalloy	156,000	0.30	Workpiece

**Table 2:** List of parameters necessary to define in order to calculate abrasive wear.

Constant	Meaning
C1	Wear coefficient, K
C2	Material hardness, H
C3	Pressure exponent, m
C4	Velocity exponent, n

**Table 3:** Material constants used in the numerical model.

Constant	Meaning	Tool
C1	Wear coefficient, K	1.7 × 10^−5^ [40]
C2	Material hardness, H	1290 [33,41]
C3	Pressure exponent, m	1 [33]
C4	Velocity exponent, n	0 [33]

**Table 4:** R factor for tests.

Test Number	R, mm
1	98.5
2	97.5
3	96.5
4	95.5
5	94.5
6	93.5
7	92.5

**Table 5:** Amount of material removed from the bristles for different engagement depths.

Test	R, mm	Depth of Engagment, mm	Rotational Speed, rpm	Quantity of Worn Material, mm^3^
1	98.5	1.5	1400	0.044
2	97.5	2.5		0.045
3	96.5	3.5		0.047
4	95.5	4.5		0.049
5	94.5	5.5		0.051
6	93.5	6.5		0.053
7	92.5	7.5		0.055

**Table 6:** Measurement values of a single bristle with calculated volume.

Value of Engagement, mm	Number of Parts	Depth, 3 mm	Depth, 5 mm	Depth, 10 mm
4.5	30	0.80	0.97	1.07
4.5	60	0.56	0.81	0.98
6.5	30	0.46	0.80	0.97
6.5	60	0.46	0.74	0.99

**Table 7:** Comparison of single-bristle volume.

Value of Engagement, mm	Number of Parts	Calculated Volume of Single Bristle, mm^3^	Loss Material, mm^3^	Loss Material Per Part Based on Test, mm^3^	Loss Material Based on FEM Calculations, mm^3^	Difference, %
4.5	30	32.20	1.06	0.035	0.049	−39%
4.5	60	30.59	2.67	0.045	0.049	−10%
6.5	30	30.34	2.92	0.097	0.053	46%
6.5	60	30.18	3.08	0.051	0.053	−3%

## Data Availability

The datasets used and analyzed during the current study are available from the corresponding author upon reasonable request.
